# Frequency-specific alterations in intrinsic low-frequency oscillations in newly diagnosed male patients with obstructive sleep apnea

**DOI:** 10.3389/fnins.2022.987015

**Published:** 2022-09-30

**Authors:** Yaping Zeng, Yongqiang Shu, Xiang Liu, Panmei Li, Linghong Kong, Kunyao Li, Wei Xie, Li Zeng, Ting Long, Ling Huang, Haijun Li, Dechang Peng

**Affiliations:** ^1^Medical Imaging Center, The First Affiliated Hospital of Nanchang University, Nanchang, China; ^2^PET Center, The First Affiliated Hospital of Nanchang University, Nanchang, China

**Keywords:** frequency band, intrinsic brain activity, resting-state fMRI, cognitive impairment, obstructive sleep apnea

## Abstract

**Purpose:**

Previous studies found abnormal low-frequency spontaneous brain activity related to cognitive impairment in patients with obstructive sleep apnea (OSA). However, it is unclear if low-frequency spontaneous brain activity is related to specific frequency bands in OSA patients. In this study, we used the amplitude of low-frequency fluctuation (ALFF) method in patients with OSA to explore characteristics of spontaneous brain activity in the classical (0.01–0.1 Hz) and five sub-frequency bands (slow-2 to slow-6) and analyzed the relationship between spontaneous brain activity and clinical evaluation was analyzed.

**Patients and methods:**

Resting-state magnetic resonance imaging data and clinical assessments were collected from 52 newly-diagnosed OSA patients and 62 healthy controls (HCs). We calculated the individual group ALFF values in the classical and five different sub-frequency bands. A two-sample *t*-test compared ALFF differences, and one-way analysis of variance explored interactions in frequency bands between the two groups.

**Results:**

ALFF values in the OSA group were lower than those in the HC group in the bilateral precuneus/posterior cingulate cortex, bilateral angular gyrus, left inferior parietal lobule, brainstem, and right fusiform gyrus. In contrast, ALFF values in the OSA group were higher than those in the HC group in the bilateral cerebellum posterior lobe, bilateral superior frontal gyrus, bilateral middle frontal gyrus, left inferior frontal gyrus, left inferior temporal gyrus, and left fusiform gyrus. Some ALFF values in altered brain regions were associated with body mass index, apnea-hypopnea index, neck circumference, snoring history, minimum SaO_2_, average SaO_2_, arousal index, oxygen reduction index, deep sleep period naming, abstraction, and delayed recall in specific frequency bands.

**Conclusion:**

Our results indicated the existence of frequency-specific differences in spontaneous brain activity in OSA patients, which were related to cognitive and other clinical symptoms. This study identified frequency-band characteristics related to brain damage, expanded the cognitive neuroimaging mechanism, and provided additional OSA neuroimaging markers.

## Introduction

Obstructive sleep apnea (OSA) is the most common sleep disorder, characterized by airway narrowing or complete

occlusion due to repeated collapse of the upper airway during sleep. This condition leads to intermittent hypoxia, hypercapnia, and sleep fragmentation. One of the most common symptoms of OSA is snoring (Rundo, 2019), but most people consider this to be normal and thus tend to ignore the condition (Veasey and Rosen, 2019). A population-based study showed that the overall prevalence of OSA in the general adult population ranged from 9% to 38% overall, being 13%–33% in men, 6%–19% in women, and much higher in older age groups (Senaratna et al., 2017). Common symptoms of OSA include daytime drowsiness, fatigue, personality changes, poor memory, inability to concentrate, and other indications. In severe cases, sleep deprivation can lead to car accidents caused by falling asleep while driving (Garbarino, 2017). Chronic sleep apnea can lead to hypertension, cardiovascular disease (Rana et al., 2020), stroke, diabetes, or premature death (Rowley et al., 2017). Neurocognitive symptoms of patients with OSA are of increasing concern to a wide range of scholars since repeated hypoxia and hypercapnia are considered to be important mechanisms of cognitive impairment in these patients. Many previous studies have reported abnormal brain outcomes and functions associated with cognition in patients with OSA; however, the mechanism of neurological damage in these patients remains unclear. Exploring the neurological mechanisms underlying OSA is crucial for diagnosis and treatment of patients.

With the rapid development of magnetic resonance imaging (MRI) equipment and technology, functional magnetic resonance imaging (fMRI) has become widely used in neuropsychiatric diseases, and an increasing number of scholars are using MRI to explore the mechanism of brain damage in OSA patients. To date, many researchers have found that OSA generally causes changes in structure (Macey et al., 2008, 2018; Chen et al., 2020), function (Park et al., 2016), and network properties (Chen et al., 2017), and these findings have contributed significantly to the understanding of brain damage in OSA patients. A previous analysis (Joo et al., 2013) showed localized cortical thinning in brain regions such as the anterior cingulate, insula, and inferior parietal lobule (IPL). In addition, the number of respiratory arousals was associated with cortical thinning in the anterior cingulate gyrus and IPL, and a visual memory test was related to the cortical thickness of the parahippocampal gyrus and uncinate gyrus suggesting, that cognitive impairment and upper respiratory tract sensorimotor disorders are associated with cortical thinning in OSA patients. A functional connectivity (FC) study (Zhou et al., 2020) showed that FC in the right hippocampus and bilateral insular, right thalamus, and right anterior cingulate gyrus was decreased in OSA patients. Moreover, FC between the left hippocampus and left anterior cerebellum was also decreased, whereas it was increased in the left hippocampus, right superior temporal, middle temporal gyrus, left posterior cingulate gyrus, and left angular gyrus. These results showed that during clinical evaluation, abnormal FC was present in the hippocampus of patients with OSA, suggesting possible biomarkers and a pathophysiological mechanism of neurocognitive impairment. The results of a brain network study (Park et al., 2022) showed that FC in patients with OSA was decreased in brain-cerebellar connections of different functional networks, whereas it was higher in the intercortical network connections of the default mode network (DMN) and prefrontal control network. These results indicate that abnormal brain-cerebellar pathways may be related to sleep fragmentation and hypoxia in OSA, and that abnormal DMN function may be associated with cognitive impairment.

The amplitude of low-frequency fluctuations (ALFF) reflects the average intensity of the low-frequency portion of the signal for each voxel in the blood oxygen level-dependent (BOLD) signal and typically represents the intensity of brain activity at a low frequency (0.01–0.08 Hz) (Cui et al., 2014; Hu et al., 2021). The BOLD signal intensity can reflect the activation and inactivation of the brain. ALFF reveals the activity of local spontaneous neurons in the brain (Zang et al., 2007), which can be used to distinguish the physiological state of internal brain disease from local brain injury in the resting state (Jiang et al., 2021). This suggests that ALFF could be a potential biomarker because of its high temporal stability (Kublbock et al., 2014). Wu et al. (2020) suggested that ALFF and regional homogeneity (ReHo) may provide better clinical applications than FC and network. Since ALFF was initially proposed (Biswal et al., 1995), it has been widely used in the examination of various neuropsychiatric diseases (Wang et al., 2019; Mu et al., 2020; Rosenbaum et al., 2020), demonstrating the utility of this method. We previously used ALFF (Li et al., 2015) and ReHo (Peng et al., 2014) methods to explore spontaneous brain activity to compare OSA patients with others and found that ReHo values were significantly lower in the right medial frontal gyrus (MFG), right superior frontal gyrus (SFG), right cluster of the precuneus and angular gyrus, and left superior parietal lobule in OSA patients. In contrast, ReHo was significantly higher in the right posterior lobe of the cerebellum, right cingulate gyrus, and bilateral cluster covering the lentiform nucleus, putamen, and insula. ALFF values were significantly lower in the right precuneus and bilateral posterior cingulate gyrus clusters in OSA patients. These findings suggest functional disorders and cognitive impairment in the default network area in OSA patients.

All the studies mentioned above were based on the classical low-frequency range (0.01–0.08 Hz) and to some extent characterized the spontaneous brain activity of patients with OSA. However, the inherent spontaneous activity patterns of the brain have recently been shown to be sensitive to specific frequency bands, and the low-frequency oscillation amplitudes of different frequency bands are thought to reflect meaningful differences between brain regions (Zuo et al., 2010). Some scholars have divided the frequency band into different sub-bands, including slow-2 (0.198–0.25 Hz), slow-3 (0.073–0.198 Hz), slow-4 (0.027–0.073 Hz), slow-5 (0.01–0.027 Hz), and slow-6 (0–0.01 Hz) (Luo et al., 2020; Li et al., 2021b). At present, the existence of frequency characteristics of spontaneous brain activity has been reported in many psychoneurological disorders, such as Alzheimer’s disease and insomnia (Wang et al., 2016; Zhou et al., 2016; Li et al., 2017), which is useful for elucidating their neural mechanisms. Recently, our group studied OSA patients before and after treatment in slow-4 and slow-5 studies based on the ReHo method (Li et al., 2021a) and found that the reversal of local spontaneous brain activity in OSA after short-term continuous positive airway pressure (CPAP) treatment was frequency dependent. This finding contributed to a better understanding of the characteristics of local neuroimaging features and may be used as a potential biomarker for clinical CPAP therapy. However, the intensity of spontaneous brain activity in all sub-bands (slow-2 to slow-6) in patients with OSA is currently unknown.

Based on the above questions, we hypothesized that frequency characteristics of spontaneous brain activity exist in patients with OSA and are associated with cognitive function. To test this hypothesis, we used voxel-level ALFF to detect local spontaneous brain activity characteristics across slow-2 to slow-6 bands in OSA patients. Second, we correlated the abnormal brain areas in different frequency bands with clinical scales to explore the physiological significance of different spontaneous brain activities.

## Materials and methods

### Subjects

A total of 56 male right-handed patients newly diagnosed with moderate and severe OSA who attended the first respiratory consultation at the First Affiliated Hospital of Nanchang University from July 2014 to January 2019 were included. The specific inclusion criteria for the OSA group were as follows: (1) age 18–65 years; (2) snoring history of at least half a year; and (3) apnea hypopnea index (AHI) ≥ 15 times/h (using the diagnostic criteria of sleep diseases of the American Academy of Sleep Medicine). The exclusion criteria were as follows: (1) other sleep-related diseases (such as primary insomnia) and psychiatric and central nervous system diseases (such as epilepsy, depression, and schizophrenia); (2) history of psychotropic and prohibited drug use or alcohol abuse; (3) contraindications or inability to tolerate MRI examination (such as claustrophobia); and (4) presence of intracranial lesions detected by routine MRI scans. Sixty-five healthy men of similar age, education, and handedness to the OSA group participants with AHI ≤ 5 times/h were recruited as healthy controls (HCs) during the same period, with similar exclusion criteria. This study has been approved by the Medical Research Ethics Committee of The First Affiliated Hospital of Nanchang University, and each participant has signed a written informed consent.

### Polysomnography and clinical scale evaluation

All subjects underwent overnight polysomnography (PSG) (Alice5 LE; Respironics, Orlando, FL, United States) to confirm the diagnosis of OSA and rule out other sleep disorders. Subjects were instructed to avoid hypnotics, alcoholic beverages, and coffee for at least 7 h prior to the test. A professional technician performed the PSG examinations and scored the results according to the guidelines of the American Academy of Sleep Medicine (AASM) (Kapur et al., 2017). PSG monitoring included EEG, EOG, EMG, snoring, postural changes, nasal airflow of breathing, chest and abdominal movements, and blood oxygenation. PSG calculates and reports the following sleep stages: awake period, light sleep period, deep sleep period (N1, N2, N3), rapid eye movement (REM), AHI, arousal index (AI), oxygen reduction index, mean oxygen saturation (SaO_2_), minimum SaO_2_, and other indicators.

We assessed all subjects on the scale on the day of the MRI. All participants were assessed on the Epworth Sleepiness Scale (ESS), a semi-objective scale used to assess daytime sleepiness. The ESS has a total score of 24, with scores > 6 indicating drowsiness, > 11 indicating excessive drowsiness, and > 16 indicating dangerous drowsiness. The Montreal Cognitive Assessment (MoCA) assesses cognitive impairment of different cognitive domains, including attention and concentration, executive function, memory, language, visuospatial, abstraction, computation, and orientation, with a total score of 30 points and a score ≤ 26 indicating mild cognitive impairment.

### fMRI data acquisition

All MRI data were acquired from the 3.0T scanner (Erlangen, Siemens, Munich, Germany) with an 8-channel phased-array magnetic head coil at the First Affiliated Hospital of Nanchang University Before scanning, the subjects were instructed to relax and close their eyes to avoid systematic thinking and falling asleep. During the scan, fixed foam was used to reduce head movement, and soft earplugs were used to reduce machine noise. All participants were initially subjected to conventional T_1_-weighted imaging [repetition time (TR) = 250 ms, echo time (TE) = 2.46 ms, thickness = 5 mm, gap = 1.5 mm, field of view (FOV) = 220 mm × 220 mm, slices = 19] and T_2_-weighted imaging (TR = 4,000 ms, TE = 113 ms, thickness = 5 mm, gap = 1.5 mm, FOV = 220 mm × 220 mm, slices = 19), in order to exclude those with obvious brain lesions. Finally we used a gradient recalled echo planar imaging (EPI) pulse sequence(TR = 2,000 ms, TE = 30 ms, flip angle = 90°, FOV = 230 mm × 230 mm, matrix = 64, thickness = 4 mm, gap = 1.2 mm) for an 8-min rs-fMRI scan, and a fast gradient recalled echo sequence(TR = 1,900 ms, TE = 2.26 ms, thickness = 1.0 mm, gap = 0.5 mm, FOV = 250 mm × 250 mm, matrix = 256 × 256, flflip angle = 9°) to obtain high-resolution three-dimensional T1-weighted 176 structural images.

### fMRI data preprocessing

Data accuracy and image quality were initially checked using Microro software^1^. The DPABI^2^ based on MATLAB R2018a (MathWorks, Natick, MA, United States) and SPM12 (Statistical Parametric Mapping 12^3^, were used for image preprocessing. DICOM format data were formatted to NIFTI. The first 10 time points were discarded due to the time required for the machine to enter a stable state and subjects to adapt to the environment, and the remaining 230 time points were corrected to the same time point in the time layer. In order to minimize the effect of head movement for three-dimensional head movement correction, the head movement criteria used were maximum directional displacement (x, y, z) < 1.5 mm and maximum rotation (x, y, z) < 1.5°, and when head movement exceeded the standard range, it was excluded. Four patient and three HCs were excluded due to excessive head motion. Normalization of all MRI functional images was to Montreal Neurological Institute standard space with an EPI template and resampling at 3 × 3 × 3 mm^3^ voxel resolution. Images were spatially smoothed with a 6 × 6 × 6 mm^3^ full width at half maximum Gaussian filter. After preprocessing, further linear regression was performed to remove interfering factors (including white matter signal, cerebrospinal fluid signal, head motion parameters, and global signal).

### Amplitude of low-frequency fluctuation analysis

Amplitude of low-frequency fluctuation was calculated using DPABI (see text footnote 2). ALFF values were calculated for each voxel’s BOLD time series fluctuations to a fast Fourier change to obtain a power spectrum. The power spectrum was then squared and the mean value was calculated as the ALFF value. To reduce the effect of different subjects, the ALFF for each voxel was divided by the whole-brain average ALFF value to produce a normalized ALFF value (Zang et al., 2007). To study the variation in different frequency bands, six different frequency bands [classical band (0.01–0.1 Hz) and five sub-bands: slow-2 (0.198–0.25 Hz), slow-3 (0.073–0.198 Hz), slow-4 (0.027–0.073 Hz), slow-5 (0.01–0.027 Hz), and slow-6 (0–0.01 Hz)] (Zhang et al., 2019; Zhou et al., 2019; Xi et al., 2020) were calculated.

### Statistical analyses

Demographic statistics and clinical indicators (body mass index [BMI], age, education, AHI, N1, N2, N3, REM, AI, ESS, minimum SaO_2_, mean SaO_2_, sleep efficiency, and MoCA scale) were statistically analyzed using the SPSS software package (version 25.0, IBM, Armonk, NY, United States). The two-sample *t*-test was used to compare the two groups, and *P* < 0.05 was considered statistically significant.

We initially performed a one-sample *t*-test to determine the ALFF spatial distribution between the two groups of subjects in the classical frequency band. We then used a two-sample *t*-test to compare the differences of ALFF values in the classical frequency band and its five sub-bands between the two groups using age and years of education as covariates. To investigate the interaction between groups and the five sub-frequency bands, one-way analysis of variance (ANOVA) analysis (flexible factorial design, 2 × 5) was performed using SPM12, with OSA and HC groups as between-group factors and different frequency bands as reproducible measures. *Post hoc t*-tests were used to compare the differences between the two groups for different frequency bands. The voxel level (*p* < 0.01) and cluster level (*p* < 0.05) were considered statistically different. The ALFF signal values of the significantly different brain regions in the two groups were saved and extracted using the REST Version 1.8 software^4^. Pearson correlation analysis was used to explore the relationship between ALFF values in different brain regions and clinical assessments.

## Results

### Analysis of demographic statistics and clinical indicators

The BMI, AHI, N1, AI, and ESS scores in the OSA group were significantly higher than those in the HC group (*P* < 0.05, FDR corrected). In contrast, the N3, minimum SaO_2_, mean SaO_2_, REM, sleep efficiency, and MoCA scores were significantly lower in the OSA group than in the HC group (*P* < 0.05, FDR corrected). Age, years of education, and N2 were not significantly different between the two groups (*P* > 0.05, FDR corrected) (Table 1).

**TABLE 1:** Demographics statistics and clinical indicators of the OSA and GS groups (X ± SD).

Category	OSA group	HC group	T value	*P* value
Age (years)	37.71 ± 9.90	39.69 ± 8.77	−1.126	0.263
Education level (years)	12.27 ± 3.04	11.41 ± 3.73	1.350	0.180
BMI (kg/m^2^)	12.69 ± 8.66	20.70 ± 1.45	−7.110	<0.001
AHI (times/h)	55.42 ± 21.91	2.48 ± 1.24	18.856	<0.001
AI (/h)	37.50 ± 22.66	11.71 ± 2.88	8.813	<0.001
minimum SaO2(%)	67.83 ± 12.53	93.20 ± 3.27	−15.233	<0.001
mean SaO2(%)	91.28 ± 4.45	96.48 ± 2.26	−7.996	<0.001
N1(%)	29.54 ± 17.11	9.95 ± 3.41	8.750	<0.001
N2(%)	39.75 ± 13.94	40.92 ± 6.26	−0.587	0.558
N3(%)	22.34 ± 17.72	29.49 ± 5.21	−3.008	0.003
REM(%)	7.93 ± 8.09	20.13 ± 6.79	−8.605	<0.001
MoCA (score)	25.10 ± 3.13	27.48 ± 1.68	−4.908	<0.001
ESS (score)	11.79 ± 4.11	3.62 ± 2.33	13.241	<0.001
Snoring history (years)	10.846 ± 7.8325	-	-	-

BMI, body mass index; AHI, apnea hypopnea index; AI, arousal index; REM, rapid eye movement; MoCA, Montreal Cognitive Assessment; ESS, Epworth Sleepiness Scale.

### Spatial distribution pattern of amplitude of low-frequency fluctuation between the two groups in the classical frequency band

**FIGURE 1:**
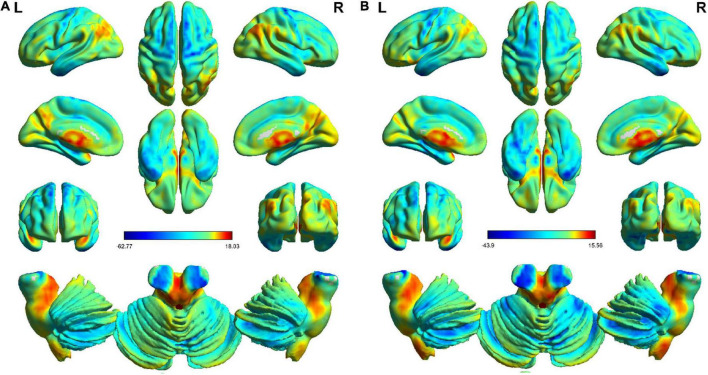
One-sample *t*-test (*P* < 0.01, FDR corrected) distribution of the results of the classical frequency bands in the HC and OSA groups. Generally speaking, the spatial distribution of the two groups was similar. Panel **(A)** is the distribution in the HC group. Panel **(B)** is the distribution in the OSA group.

A one-sample *t*-test was performed in the classical frequency band to determine the spatial distribution of ALFF, and the results showed that the two groups had similar distributions (Figure 1).

### Intergroup interactions with the five frequency bands

**FIGURE 2:**
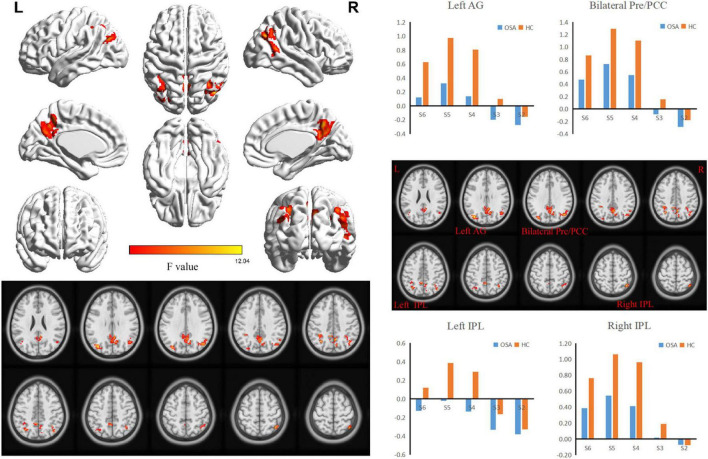
The interaction between specific frequency bands (Slow-2 to Slow-6) and groups (OSA patients and HC) was based on ANOVA (flexible factorial design, 2 × 5, two-tailed, voxel level *P* < 0.01, GRF corrected, cluster level *P* < 0.05). AG, Angular Gyrus; Pre, Precuneus; PCC, posterior cingulate cortex; IPL, Inferior Parietal Lobule.

ANOVA analysis suggested a significant interaction between OSA disease status and five specific frequency bands in the right (R) inferior parietal lobe (IPL), bilateral precuneus/posterior cingulate cortex (PCUN/PCC), left (L) angular gyrus (ANG), and L-IPL (Table 2 and Figure 2).

**TABLE 2:** Significant interaction between groups and the five specific frequency bands on ALFF (full factorial design, 2 × 5).

Brain regions	BA	MNI coordinates			F scores	Cluster size
		X	Y	Z		
R-IPL	40	42	−57	57	12.04	159
Bilateral PCUN/PCC	7,23	6	−57	42	9.92	231
L-ANG	19,39	−36	−75	30	11.96	41
L-IPL	40	−33	−63	42	10.14	53

All clusters were analyzed using a two-tailed test with a voxel-level threshold of *P* < 0.01, GRF correction, and cluster-level of *P* < 0.05. L, left; R, Right; IPL, Inferior Parietal Lobule; PCUN, Precuneus; PCC, posterior cingulate cortex; ANG, Angular Gyrus.

### Comparison of amplitude of low-frequency fluctuation differences between the classical band and five sub-bands in the obstructive sleep apnea and healthy control groups

**FIGURE 3:**
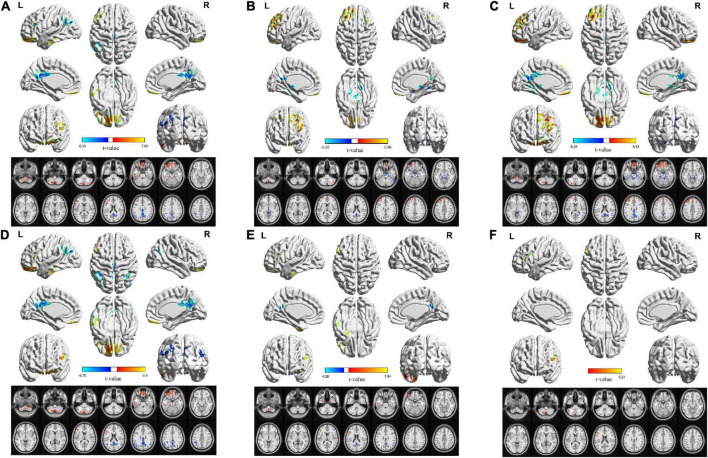
Corresponding changes in ALFF values in the classical band and the slow-2 to slow-6 band in OSA patients. **(A)** Classical band (0.01–0.1 Hz); **(B)** slow-2 (0.198–0.25 Hz); **(C)** slow-3 (0.073–0.198 Hz); **(D)** slow-4 (0.027–0.073 Hz); **(E)** slow-5 (0.01–0.027 Hz); **(F)** slow-6 (0–0.01 Hz) (double tail, voxel level *P* < 0.01, GRF corrected, cluster level *P* < 0.05).

In the classical frequency band, the L-MFG, bilateral PCUN/PCC, L-ANG, and L-IPL ALFF values in the OSA group were significantly lower than those in the HC group. In contrast, R-cerebellum posterior lobe (CPL), L-CPL, L-fusiform gyrus (FUG), L-SFG, and L-inferior frontal gyrus (IFG) ALFF values in the OSA group were significantly higher than those in the HC group (Table 3 and Figure 3A).

**TABLE 3:** Significant alterations of the ALFF of typical frequency band (0.01–0.1 Hz) between the OSA patients and HCs.

Brain regions	BA	MNI coordinates			t-scores	Cluster size
		X	Y	Z		
R-CPL	/	9	−54	−57	6.134	122
L-CPL	/	−18	−60	−57	5.245	78
L-FUG	20	−54	−18	−30	5.391	95
L-SFG	11	−45	30	−18	5.996	285
L-IFG	48	−39	18	21	7.655	65
L-MFG	9	−39	30	21	−5.495	49
Bilateral PCUN/PCC	7,23	0	−36	27	−5.505	295
L-ANG	19	−36	−75	30	−6.325	47
L-IPL	7	−33	−63	42	−5.697	112

All clusters were analyzed using a two-tailed test with a voxel-level threshold of *P* < 0.01, GRF correction, and cluster-level of *P* < 0.05. L, left; R, Right; CPL, Cerebellum Posterior Lobe; FUG, Fusiform Gyrus; SFG, Superior Frontal Gyrus; IFG, Inferior Frontal Gyrus; MFG, Middle Frontal Gyrus; PCUN, Precuneus; PCC, posterior cingulate cortex; ANG, Angular Gyrus; IPL, Inferior Parietal Lobule.

In the slow-2 band, ALFF values of the brainstem and bilateral PCUN/PCC in the OSA group were significantly lower than those in the HC group, whereas the R-CPL, L-MFG, L-SFG, and R-SFG values in the OSA group were significantly higher than those in the HC group (Table 4 and Figure 3B).

**TABLE 4:** Significant alterations of the ALFF of five specific frequency bands between the OSA patients and HCs.

Brain regions	BA	MNI coordinates			t-scores	Cluster size
		X	Y	Z		
**Slow-6 (0–0.01 Hz)**						
L-CPL	/	−15	−69	−54	5.244	83
L-IFG	48	−39	18	21	5.188	41
**Slow-5 (0.01–0.027 Hz)**						
R-CPL	/	9	−54	−57	5.533	46
L-ITG	20	−33	−3	−42	4.667	38
L-FUG	20	−54	−18	−33	5.048	47
L-IFG	11	−36	36	−21	5.007	37
Bilateral PCUN/PCC	23	6	−51	27	−4.279	45
**Slow-4 (0.027–0.073 Hz)**						
L-CPL	/	−15	−69	−54	5.475	140
R-CPL	/	9	−57	−57	6.038	197
L-FUG	20	−54	−18	−30	5.121	95
L-SFG	38	−45	30	−18	5.832	210
L-IFG	48	−42	18	21	6.798	62
Bilateral PCUN/PCC	7,23	0	−36	27	−5.511	319
L-ANG	39	−39	−75	30	−6.748	55
R-ANG	39	48	−63	27	−5.367	46
L-IPL	40	−33	−45	39	−5.916	137
**Slow-3 (0.073–0.198 Hz)**						
L-CPL	/	−18	−81	−45	5.659	63
R-SFG, MFG	11,47	21	36	−21	5.261	239
L-SFG, MFG	8,11	−15	48	39	5.852	310
Brainstem		12	−33	−30	−5.66	246
Right Fusiform	30	15	−45	−12	−4.435	68
Bilateral PCUN/PCC	7,23	6	−51	27	−6.251	246
**Slow-2 (0.198–0.25 Hz)**						
R-CPL	/	39	−69	−45	4.794	38
L-MFG		−21	45	−21	4.741	80
L-SFG		−21	48	42	5.566	263
R-SFG	8	30	30	54	5.859	84
Brainstem		9	−12	−18	−5.329	189
Bilateral PCUN/PCC	23,31	−**3**	−51	21	−4.989	35

All clusters were analyzed using a two-tailed test with a voxel-level threshold of *P* < 0.01, GRF correction, and cluster-level of *P* < 0.05. L, left; R, Right; ITG, Inferior Temporal Gyrus; ANG, Angular Gyrus; CPL, Cerebellum Posterior Lobe; FUG, Fusiform Gyrus; SFG, Superior Frontal Gyrus; IFG, Inferior Frontal Gyrus; MFG, Middle Frontal Gyrus; PCUN, Precuneus; PCC, posterior cingulate cortex; IPL, Inferior Parietal Lobule; BA, Brodmann area; MNI, Montreal Neurological Institute.

In the slow-3 band, ALFF values of the brainstem, R-FUG, and bilateral PCUN/PCC in the OSA group were significantly lower than those in the HC group, whereas L-CPL, bilateral SFG, bilateral MFG, values were significantly higher in the OSA group than in the HC group (Table 4 and Figure 3C).

In the slow-4 band, ALFF values of the bilateral PCUN/PCC, L-ANG, R-ANG, and L-IPL were significantly lower in the OSA group than in the HC group, whereas the L-CPL, R-CPL, L-FUG, L-SFG, and L-IFG values in the OSA group were significantly higher than those in the HC group (Table 4 and Figure 3D).

In the slow-5 band, ALFF values of the bilateral PCUN/PCC were significantly lower in the OSA group than in the HC group, whereas values of R-CPL, L-inferior temporal gyrus (ITG), L-FUG, and L-IFG were significantly higher in the OSA group than in the HC group (Table 4 and Figure 3E).

In the slow-6 band, ALFF values of the L-CPL and L-IFG were significantly higher in the OSA group than in the HC group (Table 4 and Figure 3F).

### Analysis of the correlation between brain areas with reduced amplitude of low-frequency fluctuation values and clinical indicators in obstructive sleep apnea patients

In the classical frequency band, the bilateral PCUN/PCC was positively correlated with minimum SaO_2_, mean SaO_2_, and naming, and negatively correlated with BMI, snoring history, AHI, microarousal index, and oxygen reduction index. The L-ANG was positively correlated with minimum SaO_2_, naming, and abstraction, and negatively correlated with sleep efficiency. The L-IPL was positively correlated with N3 and naming and negatively correlated with snoring history and delayed recall (Table 5).

**TABLE 5:** Significant correlations between the altered ALFF in different bands and clinical assessment in OSA patients.

Frequency band	Brain area	BMI	Neck circumference	AHI	Minimum SaO_2_	Mean SaO_2_	Sleep efficiency	N3	AI	Oxygen decrement index	Naming	Delayed recall	Abstraction	ESS	Snoring history
Typical frequency band	Bilateral PCUN/PCC	−0.331*	−0.197	−0.369**	0.504**	0.385**	−0.143	0.016	−0.510**	−0.405**	0.399**	−0.15	0.136	−0.245	−0.503**
	L-ANG	−0.097	−0.176	−0.135	0.343*	0.049	−0.384**	0.027	−0.207	−0.215	0.344*	0.044	0.285*	−0.164	−0.223
	L-IPL	−0.014	−0.09	0.133	0.192	0.104	−0.181	0.275*	0.054	−0.045	0.298*	−0.300*	0.073	−0.102	−0.337*
Slow-2 band	Brainstem	−0.298*	−0.302*	−0.498**	0.378**	0.319*	0.108	−0.117	−0.408**	−0.424**	0.273	−0.07	0.124	−0.136	−0.335*
	Bilateral PCUN/PCC	−0.379**	−0.273	−0.24	0.364**	0.361**	−0.069	0.087	−0.427**	−0.184	0.114	−0.162	−0.041	−0.218	−0.076
Slow-3 band	Brainstem	−0.298*	−0.300*	−0.413**	0.370**	0.329*	0.091	−0.156	−0.467**	−0.378**	0.294*	−0.132	0.109	−0.109	−0.345*
	Right Fusiform	−0.371**	−0.271	−0.498**	0.451**	0.502**	0.162	−0.097	−0.517**	−0.481**	0.191	−0.096	0.039	−0.021	−0.404**
	Bilateral PCUN/PCC	−0.346*	−0.235	−0.332*	0.396**	0.379**	−0.054	0	−0.387**	−0.336*	0.238	−0.156	0.029	−0.193	−0.313*
Slow-4 band	Bilateral PCUN/PCC	−0.299*	−0.178	−0.338*	0.525**	0.396**	−0.127	0.061	−0.478**	−0.386**	0.393**	−0.182	0.135	−0.169	−0.516**
	L-ANG	−0.144	−0.222	−0.107	0.357**	0.105	−0.402**	0.008	−0.235	−0.201	0.376**	−0.007	0.241	−0.128	−0.281*
	R-ANG	−0.109	−0.085	−0.189	0.164	0.185	−0.157	0.013	−0.278*	−0.259	0.035	−0.063	0.019	−0.102	−0.274*
	L-IPL	−0.056	−0.11	0.07	0.264	0.177	−0.124	0.245	−0.029	−0.088	0.302*	−0.346*	0.069	−0.139	−0.350*
Slow-5 band	Bilateral PCUN/PCC	−0.296*	−0.214	−0.388**	0.414**	0.329*	−0.085	−0.054	−0.504**	−0.391**	0.219	0.004	0.072	−0.361**	−0.335*

*At level 0.05 (double tail), the correlation was significant. **At level 0.01 (double tail), the correlation was significant.

In the slow-2 band, the brainstem was positively correlated with minimum SaO_2_ and mean SaO_2_, and negatively correlated with BMI, snoring history, AHI, neck circumference, microarousal index, and oxygen decrement index. The bilateral PCUN/PCC was positively correlated with minimum SaO_2_ and mean SaO_2_ and negatively correlated with BMI and the microarousal index (Table 5).

In the slow-3 band, the brainstem was positively correlated with minimum SaO_2_, mean SaO_2_, and naming, and negatively correlated with BMI, snoring history, neck circumference, AHI, AI, and oxygen reduction index, whereas the R-FUG was positively correlated with minimum SaO_2_ and mean SaO_2_, and negatively correlated with BMI, snoring history, AHI, AI, and oxygen reduction index. The bilateral PCUN/PCC was positively correlated with minimum SaO_2_ and mean SaO_2_ and negatively correlated with BMI, snoring history, AHI, AI, and oxygen reduction index (Table 5).

In the slow-4 band, the bilateral PCUN/PCC was positively correlated with minimum SaO_2_, mean SaO_2_, and naming, and negatively correlated with BMI, snoring history, AHI, AI, and oxygen decrement index. The L-ANG was positively correlated with minimum SaO_2_ and naming and negatively correlated with sleep efficiency and snoring history. The R-ANG was negatively correlated with AI, and the L-IPL was positively correlated with naming and negatively correlated with delayed recall and snoring history (Table 5).

In the slow-5 band, the bilateral PCUN/PCC was positively correlated with minimum SaO_2_ and mean SaO_2_ and negatively correlated with BMI, snoring history, AHI, AI, oxygen reduction index, and ESS (Table 5).

## Discussion

We have previously studied spontaneous low-frequency brain activity in male OSA patients (Li et al., 2015). This study focused on frequency dependence in patients with OSA, and the results were consistent with previous studies and also identified new findings. Primarily, we showed that ALFF values were decreased in OSA patients in the following areas: (1) the bilateral PCUN/PCC in all four frequency sub-bands except slow-6; (2) the L-IPL and L-ANG in the classical and slow-4 frequency bands; (3) the R-ANG in the slow-4 frequency band; (5) the brainstem in the slow-2 and slow-3 frequency bands; (6) the R-FUG in the slow-3 frequency band; and (7) the L-MFG in the classical frequency band. We also found that the bilateral PCUN/PCC, brainstem, R-FUG, and L-ANG showed significant positive correlations with minimum SaO_2_ and mean SaO_2_ values, and a significant negative correlation was found with AI and the oxygen decrement index. These results suggest that abnormal spontaneous brain activity in patients with OSA is associated with specific frequency bands and that correlations exist between some abnormal brain areas and clinical indicators of sleep and breathing. Furthermore, this study provides some limited information for understanding the underlying neural mechanisms of OSA.

The function of the PCUN is closely related to information integration processing, including visuospatial imagery, situational memory extraction, self-processing, and awareness (Cavanna and Trimble, 2006). Salla et al. (Yla-Herttuala et al., 2021) found amyloid-β deposition in the precuneus and posterior cingulum regions and low cortical glucose metabolism in these brain regions in patients with OSA, which may be an important factor contributing to later cognitive impairment. The PCC is involved in spatial processing, spatial action, memory, self-reflection, and self-imagination functions (Rolls, 2019) and plays an important role in cognitive function (Leech and Sharp, 2014). Attention-task MRI studies in OSA patients showed reduced brain activation in the cingulate region (Ayalon et al., 2009). Chen et al. (2016) showed that resting-state functional connectivity in the PCC of OSA patients was significantly lower than that in HCs, and Yun et al. (2017) found that OSA accelerates amyloid deposition in the right PCC, which may contribute to the progression toward Alzheimer’s disease. These results were similar in that both found abnormal brain activity and harmful substance deposition in PCUN and PCC in OSA patients, suggesting that OSA patients are susceptible to impaired PCUN and PCC. Both in the classical band and in the other five sub-bands, we find that the PCUN/PCC exhibited a decrease in ALFF values. The results showed that OSA patients had PCUN and PCC damage and cognitive impairment, and the results were relatively stable independent of frequency. Our results also revealed that PCUN and PCC was positively correlated with minimum SaO_2_ and mean SaO_2_, while negatively correlated with AHI, microarousal index, and oxygen decrement index. The potential causal relationship remains unclear and deserves further exploration.

The IPL plays a specific role in abstract motor information, language learning, and conscious motor function (Desmurget and Sirigu, 2012; Barbeau et al., 2017; Chen et al., 2018; Jiao et al., 2020). In a recent study, the cortical layer thickness in the L-IPL of OSA patients was significantly less than that in HCs (Joo et al., 2013). Guan et al. found that FC values in the L-IPL were higher in OSA patients. However, the values were significantly lower in the moderate-to-severe group than in the mild group, suggesting that the L-IPL in patients with moderate-to-severe OSA becomes decompensated as the disease progresses (Guan et al., 2019). Our team previously found that the voxel-wise degree centrality in the IPL of patients with OSA was significantly reduced when compared to that of HCs (Li et al., 2016a). Our results showed a positive correlation between the L-IPL and naming, which implies that OSA patients may have a naming disorder and language impairment.

The ANG is located in the posterior section of the IPL and has functions relating to semantic processing, reading and comprehension, number processing, attention and spatial cognition, memory retrieval, conflict resolution, theory of mind, and social cognition (Seghier, 2013; Bonnici et al., 2018; Humphreys et al., 2021). A transcranial magnetic stimulation study reported that the left ANG was crucial for both episode simulation and memory (Thakral et al., 2017). The ANG is recognized as a cross-modal integrated hub (van Kemenade et al., 2017). A study of pediatric OSA patients showed a decrease in ALFF values in the left ANG in children with OSA syndrome, when compared to controls (Ji et al., 2021). Another study on OSA in children from a topological perspective showed that the betweenness centrality of the left ANG decreased in patients with OSA (Luo et al., 2015). Liu et al. (2020) showed that the number of left ANG node degrees in patients with OSA was significantly lower than that in HCs. These results provide a basis for neuroimaging mechanisms and the neuropathophysiology of cognitive impairment in OSA patients. Additionally, our results suggest that the ANG is positively correlated with naming and abstraction, which may suggest a naming function in which the ANG and the IPL are jointly involved. The combined changes in the results of the classical and middle angular loop of the slow-4 bands suggest that the ANG oscillates in different frequency bands.

The PCUN, PCC, IPL, and ANG are part of the default network (Marques et al., 2018), and cortical connections and functional links exist between them (Cavanna and Trimble, 2006; van Kemenade et al., 2017). In studies of patients with OSA, simultaneous damage to multiple brain regions of the DMN is frequently seen (Joo et al., 2013; Baril et al., 2015; Li et al., 2016b). A task-state MRI study demonstrated that the DMN pattern observed in OSA patients showed more deactivated areas and reduced brain activation than that observed in HC (Ayalon et al., 2009; Prilipko et al., 2011). Another study showed that impaired cognitive function and attention in patients with OSA was associated with DMN dysfunction, and it is believed that the main factor is hypoxemia (Chang et al., 2020a). Moreover, DMN impairment has been shown to be an important factor (Cheng et al., 2020; Wang et al., 2020; Qin et al., 2021) and plays a pivotal role in cognitive function (Smallwood et al., 2021). Our study showed that multiple brain regions of the DMN were damaged, suggesting the possibility of a meridian mechanism for OSA cognitive impairment. For some abnormal brain regions, it is necessary to show damage at a specific frequency, and this will provide guidance for future OSA studies. Among the sub-bands, the IPL and ANG differed only in the Slow-4 band, suggesting a frequency dependence of the IPL and ANG. It further suggests that slow-4 may be more sensitive to detect abnormal intrinsic brain activity.

Our results showed that the slow-2 and slow-3 bands showed reduced brainstem ALFF values, and the slow-3 frequency band showed reduced ALFF values in the right cingulate gyrus, suggesting a frequency dependence in these brain regions. However, an increase in functional anisotropy and a decrease in mean diffusivity of the FUG were observed in MRI studies before and after treatment with CPAP in patients with OSA, indicating damage to the FUG in those patients (Maresky et al., 2019). The brainstem is responsible for many critical functions such as respiration, consciousness, blood pressure, heart rate, and sleep (Benarroch, 2018). Our findings show that the brainstem is associated with the minimum SaO_2_ and mean SaO_2_, indicating that it is associated with hypoxia, sleep fragmentation, and sleep disorders in OSA patients due to chronic respiratory disorders.

Sleep fragmentation and sleep disorders have been observed in patients with OSA. The proportions of REM sleep and MoCA in OSA patients were significantly different from those in HCs. The bilateral PCUN/PCC, brainstem, and R-FUG were negatively correlated with the micro-arousal index, oxygen reduction index, and AHI, whereas the ANG was negatively correlated with sleep efficiency and AI. The bilateral PCUN/PCC, brainstem, and R-FUG were correlated with the minimum SaO_2_ and mean SaO_2_. These results suggest that OSA sleep and respiratory abnormalities are associated with these brain areas. We also interestingly found that PCUN/PCC, Brainstem, ANG, and Right Fusiform were negatively correlated with snoring history, suggesting that reduced ALFF in these brain regions in OSA patients is associated with the course of the disease. This shows that the longer the duration of the disease the lower the ALFF value, which may indicate approximately severe damage to these brain regions. In this study, we demonstrated a significant interaction between OSA disease status and five specific frequency bands in the right subparietal lobule, bilateral precuneus, left angular gyrus, and left subparietal lobule, indicating that different frequency bands may have specific pathological correlation in this region.

It has been shown that low-frequency oscillatory activity is thought to be associated with neuronal fluctuations in the cortex, whereas high-frequency oscillations (> 0.08 Hz) mainly reflect white matter signals and are susceptible to interference by physiological noise (Lou et al., 2020b; Kong et al., 2022). Our results indicate that possibly brain stability is better in the slow-4, slow-5 bands and that some brain networks activated in the slow-2-slow-3 band may be dominated by white matter signals. Although the nature of these frequency-specific alterations in local neuronal homogeneity is unknown, different frequency bands should be considered in future studies of OSA to further understand the pathology of OSA.

The results of our study showed elevated ALFF values in the frontal lobe and IPL of OSA patients compared with those of HCs, suggesting an adaptive compensatory response. A single photon emission computed tomography study on cerebral perfusion (Ficker et al., 1997) found significant hyperperfusion in the frontal lobes of patients with OSA, indicating that the frontal lobe is functionally compensatory.

The present exploratory study had some limitations. First, all the subjects were male, affecting the scope of the results. Female and pediatric patients should also be included in future studies for a more comprehensive understanding of the pathogenesis of OSA. Second, diseases that might be improved by treatment can be compared before and after treatment is applied. This approach was not part of the current study. Third, we could consider studying the FC of OSA under different frequency bands. Some studies have shown an association between OSA and depression and anxiety (Harris et al., 2009; Vanek et al., 2020), and scales for these aspects could be included in future studies. Fourth, it is difficult to distinguish the edges in the image preprocessing, and some scholars suggest that it is necessary to use the fuzzy image processor based on fuzzy logic to blur the image edges and make the image results more reliable (Versaci and Morabito, 2021).

## Conclusion

In this study, frequency characteristics of abnormal low-frequency spontaneous brain activity were observed in patients with OSA using the ALFF method. Abnormal ALFF values in the default network, brainstem, and FUG were found in patients with OSA, indicating the existence of specific frequency bands in these patients. Abnormally active ALFF values in brain areas of OSA patients were associated with clinical symptoms such as cognition. These findings provide frequency-band characterization enabling the understanding of brain damage in OSA patients and extend the neuroimaging mechanisms of cognition, providing additional neuroimaging markers.

## Data availability statement

The raw data supporting the conclusions of this article will be made available by the authors, without undue reservation.

## Ethics statement

The studies involving human participants were reviewed and approved by Medical Research Ethics Committee of the First Affiliated Hospital of Nanchang University. The patients/participants provided their written informed consent to participate in this study.

## Author contributions

DP guided and designed the study. HL, YS, and YZ analyzed the experimental data. YZ analyzed the results and wrote the manuscript. XL, KL, PL, LK, WX, LZ, TL, and LH performed the data collection. HL and DP reviewed and revised the manuscript. All authors contributed to the article and approved the submitted version.

## Footnotes

1
http://www.MRIcro.com

2
http://www.rfmri.org/dpabi

3
https://www.fil.ion.ucl.ac.uk/spm/software/spm12/

4
http://www.resting-fmri.Sourceforge.net

## Abbreviations

OSA: obstructive sleep apnea
ALFF: amplitude of low-frequency fluctuation
MRI: magnetic resonance imaging
HC: healthy controls
ANOVA: analysis of variance
fMRI: functional magnetic resonance imaging
FC: functional connectivity
DMN: default mode network
BOLD: blood oxygen level-dependent
ReHo: regional homogeneity
CPAP: continuous positive airway pressure
PSG: polysomnography
EEG: electroencephalography
EMG: electromyography
EOG: electrooculography
AHI: apnea hypopnea index
AI: arousal index
ESS: Epworth Sleepiness Scale
MoCA: Montreal Cognitive Assessment
EPI: echo planar imaging
BMI: body mass index
REM: rapid eye movement
IPL: inferior parietal lobe or lobule
PCUN/PCC: precuneus/posterior cingulate cortex
ANG: angular gyrus
MFG: medial frontal gyrus
CPL: cerebellum posterior lobe
FUG: fusiform gyrus
SFG: superior frontal gyrus
IFG: inferior frontal gyrus
ITG: inferior temporal gyrus.
